# Effect of self-stigma on personal recovery: sex differences in people with psychotic spectrum disorders

**DOI:** 10.3389/fgwh.2025.1655885

**Published:** 2025-10-03

**Authors:** Daniela Leon-Morales, Jose-Blas Navarro, Maria Lamarca, Fermín González-Higueras, Pedro Torres, Jordi Cid, Eva Frigola-Capell, Irene Birulés, Susana Ochoa, Carme Vidal, Gemma Garrido, Josep Maria Crosas, Ana Aznar, Carolina Palma-Sevillano, Aina Sastre-Buades, Julia Sevilla-Llewellyn-Jones, Oscar Vallina-Fernández, Enrique Gutiérrez, Ana Calvo, ReCoMet Group, MI Aguilera, A Aldekoa, A Aznar, A Barajas, L Bassolas, I Birulés, S Blanco, C Casanovas, X Castellano, S Cazorla, J Cid, J Cobo, C Crespí, S Cristeto, JM Crosas, L Díaz, E Frigola-Capell, G Garrido, A González, F González-Higueras, A Grossi, G Guillamet, T Jimeno, A Jordà, L Jurado, M Korniyenko, I Layunta, M López-Botet, M López, T López, L Lozano, A Maestre, I Martínez, A Múñoz, N Muñoz, N Naives, JB Navarro, S Ochoa, C Palma-Sevillano, M Peniza, R Pérez, MD Piña, L Pijuan, N Puidellívol, I Ramírez, J Rico, A Sastre-Buades, A Segarra, LP Seseña, J Sevilla-Llewellyn-Jones, AL Schorr, AI Toraya, R Torras, P Torres, V Traid, O Vallina, G Vázquez, M Verdaguer, C Vidal, M Vidiella, N Zuzama, M Lamarca, Ana Barajas

**Affiliations:** 1 Department of Clinical and Health Psychology, Faculty of Psychology, Autonomous University of Barcelona, Barcelona, Spain; 2 Department of Psychobiology and Methodology of Health Sciences, Autonomous University of Barcelona, Barcelona, Spain; 3 Parc Sanitari Sant Joan de Deu, Sant Boi de Llobregat, Spain; 4 Grup MERITT, Fundació Sant Joan de Déu, Institut de Recerca Sant Joan de Déu, Barcelona, Spain; 5 Consorcio de Investigación Biomédica en Red de Salud Mental (CIBERSAM), Instituto de Salud Carlos III, Madrid, Spain; 6 Comunidad Terapéutica Jaén, Hospital Universitario de Jaén, Jaén, Spain; 7 Mental Health and Addictions Network, Institut Assistència Sanitària, Salt, Spain; 8 Mental Health and Addictions, Institut d’Investigació Biomèdica de Girona (IDIBGI-CERCA), Parc Hospitalari Martí I Julià, Edifici M2, Salt, Spain; 9 Department of Cognition, Development and Educational Psychology, University of Barcelona, Barcelona, Spain; 10 Fundació els Tres Turons, Barcelona, Spain; 11 Department of Mental Health, Consorci Sanitari de Terrassa, Barcelona, Spain; 12 Department of Mental Health, Hospital Universitari Parc Taulí, Institut D'Investigació I Innovació Parc Taulí I3PT, Universitat Autònoma, Sabadell, Spain; 13 Associacio Centre D'Higiene Mental Les Corts, Barcelona, Spain; 14 Grup COMSAL, FPCEE-Blanquerna, Universitat Ramon Llull, Barcelona, Spain; 15 Hospital de Mataró (Consorci Sanitari del Maresme), Mataró, Barcelona, Spain; 16 Department of Psychiatry, Son Llàtzer University Hospital, Palma, Spain; 17 Instituto de Psiquiatría y Salud Mental, Instituto de Investigación (IdISSC), Hospital Clínico San Carlos, Madrid, Spain; 18 Sierrallana Hospital, Cantabria Health Service, Cantabria, Spain; 19 Department of Applied Mathematics for Information and Communication Technologies, Higher Technical School of Computer Systems Engineering, Polytechnic University of Madrid (NEBULA Research Group), Madrid, Spain; 20 MIT linQ—Massachusetts Institute of Technology, Cambridge, MA, United States; 21 Personality, Assessment and Clinical Psychology Department, School of Psychology. Universidad Complutense de Madrid, Madrid, Spain; 22 Serra Húnter Programme, Generalitat de Catalunya, Barcelona, Spain

**Keywords:** recovery, self-stigma, schizophrenia, psychotic disorders, sex differences

## Abstract

**Introduction:**

Recently, there has been growing evidence on self-stigma and personal recovery in people with psychotic spectrum disorders. However, despite the influence of sex on mental health and the social component of self-stigma and recovery, the evidence regarding self-stigma, personal recovery, and sex is limited and inconsistent. This research aims to study the role of sex in the effect that self-stigma has on the personal recovery of people with psychotic spectrum disorders.

**Methods:**

A sample of 118 patients with a psychosis diagnose participated in the study (55.9% men). They were recruited from 9 clinical centers in Spain. Data were collected through the Internalized Stigma of Mental Illness and the Recovery Assessment Scale.

**Results:**

The effect of self-stigma on personal recovery differed according to the sex of the person. Specifically, in women, personal recovery decreased as self-stigma and alienation increased. Also, a higher self-stigma was associated with a lower personal confidence, hope and symptom control. In contrast, in men, a higher alienation was associated with higher personal confidence, hope and success orientation. These results were adjusted for educational level, comorbidity, number of psychotic episodes, and the time between symptom onset and treatment initiation.

**Discussion:**

These findings highlight the urgent need to explore further the role of sex on recovery and to have a sex-sensitive approach in policies and interventions in this population. This would benefit their recovery and, in consequence, their quality of life. Future studies should expand the sample and explore other factors that could be influencing the process of recovery and self-stigma.

## Introduction

In the last decades, there has been a growing interest in the study of the effects of stigma on people with severe mental illness. Recent studies have shown the important consequences that stigma has on numerous areas of their life and the different manifestations it may have depending on the context (1, 2). The social stigma refers to the acceptance and reproduction of stereotypes and discriminatory behavior by social majority (3, 4). On the other hand, the structural stigma manifest in the laws, policies and practices within institutions that systematically restrict the rights and access to opportunities of stigmatized groups, in this case, people with mental illness (2, 5). Lastly, since people with a mental health diagnosis are constantly exposed to social and structural stigma, they frequently internalize the stereotypes, incorporating the ones socially related to their diagnosis as a fundamental part of their identity and behave accordingly to them (6). This phenomenon is called self-stigma, and it has serious repercussions for people with mental health diagnosis.

Specifically, people with psychotic spectrum disorders are extremely affected by stigma (3). Due to social stigma, their diagnosis is frequently associated to violence, aggressiveness and unpredictability, causing an unjustified perception of them as dangerous (7). In different studies, this population has shown higher levels of self-stigma and, in association with this, lower self-esteem, neurocognitive performance and treatment adherence, and higher symptom severity, among others (8–11). In consequence, self-stigma has become a key obstacle to clinical and functional outcomes of people with psychosis, as well as their personal recovery, and, therefore, their quality of life (1, 12–15). Even, the awareness of the illness of the person, which has previously been shown to be associated with a greater adherence to treatment, when accompanied by high levels of self-stigma, have shown to increase depressive symptomatology and lower the hope and quality of life (16, 17).

In this line, the approach of recovery has recently been changing and the attention to define it has shifted from medical criteria, such as the remission of symptomatology, to psychological and phenomenological aspects, such as empowerment, self-esteem and satisfaction with one's life. Personal recovery has been described as an individualized and nonlinear process that implies the development of hope, optimism and empowerment over one's life and that is enhanced by the establishment of meaningful relationships and the sense of community. Bellack highlights that, in people with schizophrenia, this would bring a different perspective to the table that leaves behind the dooming sensation usually related to the diagnosis, chronically conditioning their identity, giving them the hope of improvement (18). This would be crucial for help-seeking and treatment adherence (19). Since self-stigma has serious consequences for personal recovery, harming the quality of life of people with psychotic spectrum disorders, a more comprehensive understanding of this interplay is essential (15, 18–20).

In this regard, it is known that men and women have different manifestations of psychosis (21). Furthermore, psychological interventions that are sensible to specific sociodemographic characteristics have been encouraged (22). Despite this, when it comes to self-stigma and personal recovery, results that consider sex differences have not been consistent to date (1, 13, 23). Considering this context, this research aims to study the role of sex in the effect that self-stigma has on personal recovery of people with psychotic spectrum disorders. We expect that the sex will have a moderation effect in the relationship between self-stigma and personal recovery. Also, we hypothesize that the effect that self-stigma has on personal recovery will be magnified in women.

## Materials and methods

### Participants

The sample included individuals with a diagnosis of a psychotic spectrum disorder who were participating in a randomized clinical trial (Identifier: NCT06423651) that included 3 different assessment moments: basal, post-treatment and 6-month follow-up. For the purpose of this study, only data from the basal moment was used. All the participants received outpatient mental health treatment from one of the following institutions: *UGC Salud Mental (Jaén), Fundació´ els Tres Turons (Barcelona), Asociación Centro de Higiene Mental Les Corts (Barcelona), Consorci Sanitari de Terrassa (Barcelona), Corporació´ Sanitària Parc Taulí (Barcelona), Consorci Sanitari del Maresme (Barcelona), Parc Sanitari Sant Joan de Déu (Barcelona), Institut d'Assistència Sanitària (Girona), Hospital Universitario Son Llatzer (Mallorca).* The inclusion criteria were: (a) age from 16 to 55 years; (b) according to the DSM-5, having a diagnostic of schizophrenia, schizophreniform disorder, delusional disorder, brief psychotic disorder, schizoaffective disorder and non-specified schizophrenia spectrum disorder or other psychotic disorders; (c) the presence of a cognitive deficit, as evidenced by a T-score below 40 in at least one of the tests administered during the pre-inclusion battery (see the Measures section); (d) obtaining through the past 6 months a score equal or greater than 4 in any of the following items of the PANSS scale: delusions, conceptual disorganization, hallucinatory behavior, suspiciousness, blunted affect, emotional withdrawal, lack of spontaneity and flow of conversation and unusual thought content; (e) be in a stable clinical phase (without psychiatric hospitalization in the last 3 months); (f) have good adherence to pharmacological treatment. On the other hand, exclusion criteria included: (a) obtaining a score equal to or greater than 5 in hostility and lack of cooperation, and equal to or greater than 6 in suspiciousness on the PANSS scale; (b) the presence of an additional diagnosis of severe disorder related to substances; (c) the presence of a brain injury, dementia or intellectual disability; (d) having participated in a cognitive rehabilitation and/or metacognitive training in the year before the clinical trial. It is important to highlight that this was the criteria used for the randomized clinical trial this study was part of.

The data analyzed in this study originate from a clinical trial for which the initial sample size was carefully calculated. The calculation was based on data published by Ochoa et al. (61), which reported a mean difference of 5.73 points (SD = 11.51) on the GAF scale between baseline and six months post-intervention. Using these figures, the required sample size was estimated with a significance level (alpha) of 0.05 and a statistical power of 0.8. Accounting for an anticipated 20% loss to follow-up, the final calculated sample size was 160 participants, evenly distributed between the two experimental arms: the Cognitive Rehabilitation Programme (Rehacop) combined with Metacognitive Training vs. Metacognitive Training alone. However, the available data on self-stigma in the present manuscript corresponded to 118 patients. The statistical power of the t-test to detect as significant differences in personal recovery total score (RAS24 total) was calculated. With the standard deviations and sample size for each sex showed in Table 3, differences between sexes of 1.45 points would be detected as significant with a power of 0.80.

### Measures

To assess compliance with the inclusion and exclusion criteria, the following tools were employed: (a) Positive and Negative Syndrome Scale (PANSS) (24, 25) to assess the severity of psychotic symptoms; (b) a cognitive battery to assess the presence of cognitive deficits in the domains of learning and verbal memory, attention, executive functions, working memory, and processing speed. The cognitive test battery administered was as follows: Test de Aprendizaje Verbal Complutense (TAVEC) (26, 27), Continuous Performance Task—Identical Pairs (CPT-IP) (28, 29), Word Accentuation Test (TAP) (30, 31), Trail Making Test (TMT) (32, 33), Weschler Adult Intelligence Scale IV (WAIS-IV) (34), Stroop Color and Word Test (Stroop) (35, 36), Wisconsin Card Sorting Test (WSCT) (33, 37); and (c) medical records.

A questionnaire generated by researchers to collect the following sociodemographic and clinical data: center of affiliation, sex, birth date, place of birth, marital status, educational level, living arrangement, number of siblings and birth order, area where they grew up, employment status, the duration of untreated psychosis, date of the treatment start, date of the first diagnosis, total number of psychotic episodes, number of previous hospitalizations, history of suicide attempts, family psychiatric history, substance use, current pharmacological and psychological treatment, diagnosis (evaluated the referring clinician, using the Diagnostic and Statistical Manual of Mental Disorders, Fifth Edition), comorbidity, physical illness associated, description of the menstrual cycle throughout life and satisfaction with treatment.

Self-stigma was measured with the Internalized Stigma of Mental Illness (ISMI) scale (38) adapted to Spanish population (39). This is a self-report scale that has 29 items rated on a 4-point Likert scale (from 1: “Totally disagree” to 4: “Totally agree”). A total score and five subscales are derived: Alienation (items from 1 to 6), Stereotype endorsement (items from 7 to 13), Perceived discrimination (items from 14 to 18), Social withdrawal (items from 19 to 24) and Stigma resistance (items from 25 to 29). The scale scores are generated as the mean of answers to their items, taking into account that the Stigma Resistance must be reverse-scored. A higher score indicates a higher self-stigma. Lysaker, Roe and Yanos (40) proposed that a score of 2 or less would indicate a “minimal stigma”; a score greater than 2 but less than 2.5, a “mild stigma”; from 2.5 to 2.9, a “moderate stigma”, and from 3 to 4, “severe stigma”. The scale showed a high internal consistency within the sample, with a Cronbach's *α* = .94 for the total score, and. 88, .88, .86, .90 and .77, respectively, for the five subscales.

Personal recovery was assessed by an adaptation (41) of the Recovery Assessment Scale (42). This is an abbreviated version (RAS-24) that includes 24 items rated on a 5-point Likert scale, where (from 1: “Totally disagree” to 5: “Totally agree”). A total score and five dimensional scores can be calculated as the sum of their items: Personal confidence and hope (items 21 and from 7 to 14), Willingness to ask for help (items from 18 to 20), Goal and success orientation (items from 1 to 5), Reliance on others (items 6, and from 22 to 24) and No domination by symptoms (items from 15 to 17). Higher scores indicated a better personal recovery. The total (Cronbach's *α* = .95) and the five dimensions had an adequate internal consistency when tested in the sample (.90, .87, .89, .78 and .82 respectively).

### Procedures

Participants were recruited by their mental health referent from the service they were attending to. Inclusion and exclusion criteria were reviewed by the clinical referent, except for the cognitive deficit criterion, which was assessed by a blinded evaluator during a two-hour session. The baseline data analyzed in this study were self-reported by the patients during a one-hour session. Prior to the assessment, the objectives and procedures of the clinical trial were explained, and participants were presented with and signed the informed consent form. The data received by the researchers was anonymous.

### Statistical analysis

The statistical analysis was done with Stata 18 and assuming a 5% type I error. First, a descriptive analysis of sociodemographic and clinical characteristics using classical and robust indexes was made. Then, the mean scores for total and scale scores of self-stigma and personal recovery were compared between sexes through Student's *t*-test. Effect size was calculated using Cohen's d and interpreted as usual (*d* ≈ 0.2 small effect, *d* ≈ 0.2 medium effect, *d* ≈ 0.8 large effect) (43).

To determine if sex moderated the relationship between self-stigma and personal recovery, separate linear regression models using the total recovery score and its five subscales as dependent variables were estimated. For each dependent variable, a model with total self-stigma, sex, and the full interaction term (Total Self-Stigma × Sex) as predictors was first tested. Then a second model that replaced the total self-stigma score with its five subscales, including their respective interaction terms with sex, was also tested. If an interaction term was significant, the marginal effects of the relevant self-stigma variable were calculated for men and women. If an interaction was not significant, it was removed from the model, and only the main effects were examined.

To determine the most suitable set of adjusting variables, a wide range of sociodemographic, clinical, and neurocognitive measures was initially considered. For each potential confounder, we calculated both the raw and adjusted regression coefficients for the model predicting total recovery from the total stigma score, sex, and their interaction. If the change in the interaction coefficient after adjustment was greater than 10%, the variable was retained as a confounder in the regression models (44, 45). Otherwise, it was discarded. Ultimately, all regression models were adjusted for level of education, comorbidity, number of psychotic episodes, and the duration of untreated psychosis. There were no signs of multicollinearity between the four adjusting terms as the maximum Spearman correlation value was 0.32.

## Results

### Sociodemographic and clinical characterization

Table 1 presents the sociodemographic, clinical and neurocognitive characteristics of the 118 participants, separately by sex. Most men had not completed mandatory education (56.1%), while the majority of women had (53.8%). The majority of participants of both sexes were pensioners or incapacitated (78.8% of men; 58.8% of women) and single (80.3% of men; 76.9% of women).

**Table 1:** Sociodemographic and clinical characteristics.

Characteristics	*N* (%) *M* (SD) [Min Md Max]	
	Male (*N* = 66)	Female (*N* = 52)
Sociodemographic		
Age	41.5 (9.2) [22, 42, 55]	39.5 (10.5) [19, 41, 55]
Educational level		
Unfinished mandatory	37 (56.1%)	16 (30.8%)
Finished mandatory	25 (37.9%)	28 (53.8%)
University	4 (6.0%)	8 (15.4%)
Occupational status		
Employed or student	4 (6.1%)	10 (19.6%)
Unemployed	10 (15.1%)	7 (13.7%)
Pensioner or incapacitated	52 (78.8%)	30 (58.8%)
Other	0 (0.0%)	4 (7.8%)
Marital Status		
Single	53 (80.3%)	40 (76.9%)
Married or live-in partner	8 (12.1%)	7 (13.5%)
Separated or divorced	5 (7.6%)	5 (9.6%)
Clinical		
Duration of untreated psychosis		
<3 months	48 (72.7%)	35 (67.3%)
3–6 months	8 (12.1%)	5 (9.6%)
6–12 months	2 (3.0%)	4 (7.7%)
>12 months	8 (12.1%)	8 (15.4%)
Number of psychotic episodes	3.3 (2.4) [1, 2.5, 12]	2.9 (2.3) [1, 2.5, 15]
Number of previous hospitalizations	2.6 (2.3) [0, 2, 12]	1.7 (1.7) [0, 1, 6]
Suicide attempts (Yes)	15 (22.7%)	15 (28.9%)
Family psychiatric history (Yes)	28 (42.4%)	26 (50.0%)
Substance use (Yes)	29 (43.9%)	16 (30.8%)
Diagnosis		
Schizophrenia	40 (61.5%)	23 (44.2%)
Schizophreniform disorder	1 (1.5%)	1 (1.9%)
Delusional disorder	0 (0.0%)	4 (7.7%)
Schizoaffective disorder	9 (13.9%)	13 (25.0%)
Brief psychotic disorder	0 (0.0%)	1 (1.9%)
Schizophrenia spectrum disorder	15 (23.1%)	10 (19.2%)
Comorbidity (Yes)	16 (24.2%)	12 (23.1%)
Physical illness associated (Yes)	24 (37.5%)	16 (30.8%)
Satisfaction with treatment (Yes)	44 (81.5%)	45 (90.0%)

*N*, number of participants; %, percentage; M, mean; SD, standard deviation; Min, minimum; Md, median; Max, maximum.

Clinically, about a quarter of participants had a history of suicide attempts, and half of the women had a family history of psychiatric disorders. A high rate of substance use was observed (43.9% of men). The most frequent primary disorder was schizophrenia (61.5% of men; 44.2% of women), and about a quarter of participants presented with psychiatric comorbidity. Satisfaction with treatment was very high in both sexes.

While the majority of participants presented with a duration of untreated psychosis of less than three months (72.7% of men; 67.3% of women), a significant minority had gone untreated for more than a year (12.1% of men; 15.4% of women). The mean number of psychotic episodes was close to value 3, and the mean number of previous hospitalizations was slightly lower (2.6 in men; 1.7 in women).

Table 2 shows the descriptive statistics for the neurocognitive measures, presented separately by sex. With the exception of the estimated premorbid IQ, all measures are summarized as T-scores. Mean scores for both sexes on the Word Accentuation Test (WAT) were close to 100, reflecting preserved premorbid intelligence. Scores for verbal memory (CVLT: California Verbal Learning Test) and sustained attention (CPT-IP: Continuous Performance Test—Identical Pairs) were below a T-score of 40, indicating a deficit that was particularly pronounced in males. In terms of cognitive flexibility, T-scores below 40 are observed in both attentional flexibility measures (such as the Trail Making Test, TMT-B) and in more complex cognitive flexibility tasks involving set-shifting (such as the Wisconsin Card Sorting Test, WSCT), with men also showing poorer performance. However, results from the STROOP test, with mean scores slightly above *T* = 50, suggest adequate inhibitory control, with similar performance for men and women. The results of the subtests from the Wechsler Adult Intelligence Scale, Fourth Edition (WAIS-IV), show mild deficits in visuospatial reasoning and processing speed, with minimal differences between sexes.

**Table 2:** Neurocognitive characteristics.

Characteristics	*M* (SD) [Min, Md, Max]	
	Male (*N* = 66)	Female (*N* = 52)
Neurocognitive variables		
WAT		
Estimated Premorbid IQ	98.7 (10.0) [75, 100, 114]	103.4 (8.7) [79, 104, 116]
CVLT (T-score)		
Immediate Recall	34.5 (8.6) [20, 34, 69]	38.2 (7.4) [21, 39, 54]
Total (Trials 1–5)	39.8 (26.2) [2, 33, 100]	42.5 (22.3) [13, 37, 100]
Short-Term Free Recall	33.9 (10.4) [12, 33, 59]	39.4 (10.8) [12, 43, 60]
Long- Term Free Recall	33.2 (11.5) [5, 33, 56]	40.1 (12.3) [10, 39, 63]
CPT-IP (T-score)		
d' index (average of 2-,3-,4-digit conditions)	33.3 (10.9) [3, 33, 59]	36.7 (11.2) [20, 36, 65]
TMT (T-score)		
TMT-A	40.5 (11.9) [17, 40, 67]	44.7 (10.5) [27, 47, 70]
TMT_B	39.5 (11.3) [17, 37, 67]	42.5 (11.6) [20, 42, 70]
WCST (T-score)		
Total errors	38.8 (9.5) [20, 40, 57]	39.8 (9.7) [20, 39, 59]
Perseverative errors	40.6 (7.3) [28, 39, 55]	40.3 (8.9) [23, 37, 59]
Non-perseverative errors	39.4 (9.5) [20, 39, 63]	41.2 (8.9) [20, 41, 57]
STROOP (T-score)		
Stroop Interference	52.5 (9.4) [22, 52, 76]	51.7 (9.5) [32, 52, 80]
WAIS-IV (T-score)		
Forward Digits	41.1 (11.0) [14, 40, 89]	43.3 (7.5) [23, 44, 57]
Backward Digits	41.8 (11.2) [24, 42, 100]	41.2 (7.5) [24, 43, 58]
Coding	36.8 (8.2) [23, 37, 53]	38.4 (8.2) [23, 37, 57]
Similarities	40.7 (12.0) [20, 40, 80]	40.8 (8.1) [20, 40, 53]
Visual Puzzles	35.4 (11.1) [20, 32, 63]	35.9 (12.3) [20, 33, 70]
Arithmetic	40.9 (10.6) [20, 40, 60]	40.2 (8.2) [20, 40, 53]

*M*, mean; SD, standard deviation; Min, minimum; Md, median; Max, maximum.

CPT-IP, continuous performance test—identical pairs; CVLT, california verbal learning test; IQ, intelligence quotient, TMT, trail making test; WAIS-IV, wechsler adult intelligence scale-fourth edition; WAT, word accentuation test; WSCT, wisconsin card sorting test.

### Self-stigma, personal recovery and sex

Table 3 shows the means and standard deviations of the total and scale scores for self-stigma (ISMI) and personal recovery (RAS-24), obtained for the whole sample and separately by sex. The total mean score for self-stigma (66.5) was slightly below the midpoint of the possible score range (72.5). The five self-stigma scales showed a similar trend, with mean values equal to or slightly lower than the midpoints of their respective possible ranges. Regardless that there were no significant differences, men showed higher scores on the self-stigma total score and all the subscales, except in Social withdrawal. In contrast, the mean total recovery score (81.8) was moderately high compared to the midpoint of its possible range (72). The mean scores for the five recovery scales were also greater than their respective midpoints, except for the “No domination by symptoms” scale, which had a mean value nearly equal to the midpoint of its possible range. Also, following the trend seen in the self-stigma scale, the mean values of total and scale scores for self-stigma and recovery were very similar for both sexes and did not differ significantly.

**Table 3:** Descriptive and sex comparison of total and subscale scores for self-stigma and recovery.

Scales	Total *M* (SD)	Men *M* (SD)	Women *M* (SD)	*p*	|*d*|
ISMI Total (29–116)	66.5 (16.9)	67.9 (16.5)	64.6 (17.4)	.288	0.20
ISMI Stigma resistance (5–20)	12.5 (3.4)	12.8 (3.4)	12.1 (3.3)	.213	0.23
ISMI Perceived discrimination (5–20)	11.4 (3.6)	11.7 (3.8)	11.0 (3.4)	.295	0.20
ISMI Social withdrawal (6–24)	13.4 (4.6)	13.3 (4.7)	13.5 (4.5)	.790	0.05
ISMI Stereotype endorsement (7–28)	14.3 (4.8)	14.6 (4.8)	13.9 (4.9)	.444	0.14
ISMI Alienation (6–24)	15.0 (4.8)	15.5 (4.5)	14.3 (5.1)	.194	0.24
RAS-24 Total (24–120)	81.8 (1.7)	83.7 (2.3)	79.3 (2.6)	.211	0.23
RAS-24 Personal confidence and hope (9–45)	29.2 (0.7)	30.4 (0.9)	27.6 (1.1)	.059	0.35
RAS-24 Willingness to ask for help (3–15)	11.4 (0.3)	11.3 (0.4)	11.5 (0.4)	.747	0.06
RAS-24 Goal and success orientation (5–25)	17 (0.6)	17.5 (0.6)	16.3 (0.7)	.161	0.26
RAS-24 Reliance on others (4–20)	14.9 (0.3)	14.9 (0.6)	14.8 (0.5)	.797	0.05
RAS-24 No domination by symptoms (3–15)	9.4 (0.3)	9.6 (0.4)	9.2 (0.4)	.514	0.12

The minimum and maximum possible score of each subscale is in brackets.

*M*, mean; SD, standard deviation; *p*, significance level; |*d*|, absolute value of Cohen's *d* (effect size).

When applying the interpretation method proposed by Lysaker et al. (40), only the scores obtained from the Stigma resistance subscale, from the whole sample (2.5) and men (2.6), and the Alienation subscale, from the whole sample (2.5) and men (2.6), were within the “moderate” level.

Table 4 shows the results of the regression models used to assess the interaction effect between self-stigma and sex on recovery. For conciseness, only results for significant interactions are presented. Our analysis confirmed that sex moderated the effect of self-stigma on personal recovery. Specifically, there were sex differences in the effect of the total self-stigma score on the total recovery score (*p* = .045) and on two specific recovery dimensions: Personal confidence and hope (*p* = .045) and No domination by symptoms (*p* = .032). In women, a higher self-stigma total score was significantly associated with lower personal recovery, both in total (*B* = −0.31; *p* = .032) and on the Personal confidence and hope (*B* = −0.15; *p* = .012) and No domination by symptoms dimensions (*B* = −0.05; *p* = .039). In contrast, in men, self-stigma did not significantly influence recovery.

**Table 4:** Significant self-stigma by sex interaction effects on personal recover*y.*

	ISMI by sex	ISMI effect		
	*F_df1, df2_ (p)*	*B*	CI 95%	*p*
RAS-24 total score				
ISMI Total	F_8,109_ = 2.07 (.045)			
Men		0.09	−0.18 to 0.35	.528
Women		−0.31	−0.60 to −0.03	.032
ISMI Alienation	F_12,105_ = 2.43 (.008)			
Men		0.89	−0.07 to 1.86	.067
Women		−1.00	−1.96 to −0.06	.037
RAS-24 Personal confidence and hope				
ISMI Total	F_8,109_ = 2.07 (.045)			
Men		0.01	−0.10 to 0.12	.827
Women		−0.15	−0.27 to −0.03	.012
ISMI Alienation	F_12,105_ = 2.52 (.006)			
Men		0.60	0.09 to 1.12	.022
Women		−0.21	−0.70 to 0.28	.399
RAS-24 Goal and success orientation				
ISMI Alienation	F_12,105_ = 2.07 (.025)			
Men		0.35	0.02 to 0.69	.040
Women		−0.01	−0.33 to 0.31	.945
RAS-24 No domination by symptoms				
ISMI Total	F_8,109_ = 2.21 (.032)			
Men		0.02	−0.02 to 0.06	.359
Women		−0.05	−0.09 to −0.01	.039

All the results are adjusted by educational level, comorbidity, number of psychotic episodes and duration of untreated psychosis.

F_df1, df2_, F value of the model including the interaction ISMI score by sex; *p*, significance level of the interaction ISMI score by sex; B, regression coefficient; CI, confidence interval.

Regarding the self-stigma subscales, the only significant interaction found was between the Alienation subscale and sex. This interaction was significant when predicting the total recovery score (*p* = .008), the Personal confidence and hope score (*p* = .006), and the Goal and success orientation score (*p* = .025). Further analysis of this interaction revealed that, for women, a higher Alienation score was significantly associated with a lower total recovery score (*B* = −1.00; *p* = .037). In contrast, for men, a higher Alienation score was significantly associated with higher scores on both the Personal confidence and hope (*B* = 0.60; *p* = .022) and the Goal and success orientation dimensions (*B* = 0.35; *p* = .040).

Our analysis also identified significant main effects that were not moderated by sex. Specifically, a higher score on the Stigma resistance subscale was associated with lower scores on both the Willingness to ask for help dimension (*B* = −0.19; *p* = .038) and the Reliance on others dimension (*B* = −0.22; *p* = .037).

## Discussion

Considering the gap of information regarding the effect of sex on self-stigma and personal recovery, this study aimed to explore the role of sex in the effect that self-stigma has on personal recovery of people with psychotic spectrum disorders. We hypothesized, first, that sex would have a moderating role in the relationship between self-stigma and personal recovery and, second, that the effect that self-stigma has on personal recovery would be greater for women. Our findings confirmed both hypotheses, showing that women suffer more pronounced repercussion from self-stigma than men regarding their personal recovery.

Dubreucq et al., found that women had a worse personal recovery than men on a cross-sectional study (46). We could hypothesize that this difference was accentuated by the effects of self-stigma on personal recovery in women. Our results show that only in women a higher self-stigma is significantly associated with a lower personal recovery. On the other hand, Hoeksema et al. found that, when it comes to long term recovery, the outcomes presented in women were better than in men. In some cases, when specifically exploring the personal recovery, the differences between both sexes were not significant (23). This highlights the urgent need to explore the sex-differences on personal recovery on the long run and the possible factors that could be interfering on this dynamic, including self-stigma.

Since there is no consistent evidence on the influence of sex on self-stigma, this research is an important contribution to the study of this dynamic in this population (1, 13, 47, 48). Carter et al. (21) show that psychotic spectrum disorders manifest differently according to the sex of the person: men tend to present more negative symptoms, while women present more affective symptoms and a greater functionality. It is fundamental to be aware that the experience of the illness and recovery is completely conditioned by the sex of the person, since the societal norms, expectations and demands vary from men to women, as part of the social construction of the genders (21, 49). Boysen et al. indicate that stereotypes from different aspects of the person intersect (50). Therefore, the finding that women's recovery appears to be more affected by self-stigma could be related to the fact that psychotic spectrum disorders are socially associated with violence, emotional instability, and unpredictability. This is opposed to the social demands and expectations for women, to be more empathic and nurturing, and less dominant (51). And so, it is possible that when women try to incorporate the social expectations of both aspects into their identity, this creates a cognitive dissonance and, in consequence, an important psychological distress and feelings of hopelessness and disempowerment. Emotionally, this could feel like a double grief, because not only are they internalizing the stereotyped characteristics of their mental illness, but they are also internalizing the idea that these will prevent them from reaching the social standards set for them.

Our findings also show that, in women, a higher score on self-stigma is associated with a lower score on the Personal confidence and hope dimension, which refers to the perception of the person's future and their ability to manage stressful situations, and No domination by symptoms, which values whether a person does not center their life on the symptomatology of the illness (41). On this regard, Corrigan et al. explained that the “why try” effect happens when people with a diagnosis stop trying to overcome the adversity of mental illness because of the intense hopelessness, low self-esteem and the changes in their behavior that self-stigma causes (52). Considering these implications of self-stigma on confidence, hope and self-esteem, it seems completely expectable that women who go through this, lose hope on their future and their capability to cope with possible obstacles. Associating this with the additional harm of the idea that they will not be able to fulfill the societal expectations, we can hypothesize that the repercussions will be greater, and that personal recovery will be even harder to achieve. In this regard, there has been findings that indicate that women with mental disorders tend to have more psychological distress, depressive symptoms, suicide attempts and anticipation of discrimination (21, 49, 53). Furthermore, Rudman et al. explain that when a woman does not meet the social standards related to their sex, they tend to be more socially punished than men (54). In addition, Pfeiffer and In-Albon found that women with mental health problems tend to receive more blame and minimization (48).

About the subscales of self-stigma, only Alienation showed a significant interaction with sex. This subscale relates to the feeling of shame, not belonging, and diminished self-esteem. In women, a higher alienation predicted a lower recovery (1, 39). This could be because, as explained, the characteristics stereotypically related to the psychotic spectrum disorders contrast with the social expectations for women (7, 51). Consequently, this would affect drastically the way they view themselves as an individual, but also as a part of society: since they do not fit into its demands, they don't belong. Regarding men, a higher score on Alienation predicted a higher score on Personal confidence and hope, and on Goal and success orientation, which values the aspirational aspect of the person and their confidence in achieving their goals (41). This would be an interesting finding since it implies that, in men, shame and a reduced self-esteem would relate with a better perception of the person's abilities, specially to cope with stress, and hope for the future. It contradicts various studies that show that higher scores in the subscales of self-stigma are associated with lower hope, empowerment, and self-efficacy (12, 15, 20, 55). According to Renström (51) men are seen and expected to be less communal, so we could hypothesize that this could be related to the fact that having a lower sense of belonging, since it is a socially assumed characteristic for them, did not imply damage to their self-perception. In addition, since they are seen and expected to be more agentive and effective on problem solving, this could be influencing on their high view on these skills during stressful situations, the process of persecuting their goals and, therefore, the hope they have for their own future (51, 56). On the other hand, it is also a possibility that the enhancement of self-perception and goal orientation are compensatory mechanisms that men develop in response to the social disconnection that alienation implies. In relation to this, different studies have shown that, when men that endorse gender demands, such as being controlled and self-sufficient, develop a mental health problem, they can tend to be more self-reliant to avoid delegating control of their lives by seeking help or relying on others (57–59). Nonetheless, the necessity to explore further this dynamic is clear. It is possible that there are underlying mechanisms and other factors that remain unknown in our models that influence these results. It is important to mention that literature on these specific dimensions is scarce.

In addition, it is important to highlight that, despite not having a significant interaction, the Social withdrawal subscale got a considerably low *p*-value. This could be either because other covariables would fit better into this interaction model, or because the sample size didn't have enough statistical power to show significant interaction. It would be important to explore this interaction further with a bigger sample and to consider different covariables. This could be explained in relation to the “why try” effect and the alienation subscale (52): if women don't feel like they belong in a community, due to not being able to reach its standards, it is expectable that they stop trying to interact and fit, withdrawing themselves from society and, therefore, from the possibility of social support, but also, social stigma. On consonance with this, Khan et al. obtained significant results on this dimension when comparing them between men and women from Pakistan (53). We could infer from this that the sociocultural aspects might play a role in this dynamic, differing according to the place where one is born or where one lives.

We did not find significant results on the Stigma resistance subscale of self-stigma, which refers to the capacity to overcome, question and resist social stigma (3, 39). This is consistent with the findings of Dubreucq et al., who explored the moderating effect of gender on the relationship between stigma resistance and personal recovery, found that gender did not play a moderating role in this relationship (60). Nevertheless, we found that, when sex was not considered, a higher score on Stigma resistance was associated with a lower score on Willingness to ask for help and Reliance on others. In relation to this, Dubreucq et al. found that a higher stigma resistance was associated with a lower insight, which could be having a protecting effect against the stigma manifested in social interaction (60). Taking this into account, it may be possible that the lack of disposition to ask for help and rely on others is due to the absence of awareness of the illness. This, at the same time, could be contributing to the stigma resistance since the person would not be completely aware of the implications of this stigma in their life. It would be interesting to expand the research of these constructs exploring in addition the role that the insight may have.

Also, there were not significant results regarding the subscales of Stereotype endorsement and Perceived discrimination. In contrast to the latter, Khan found significant results when comparing men and women from Pakistan on the Perceived discrimination subscale (53). This difference could be based on cultural or sociodemographic aspects of the samples, but it would be important to verify this postulation.

This study highlights the urgent need to develop sex-sensitive public policies and interventions, considering the different effects of self-stigma between men and women. This would bring an important benefit for the personal recovery, specially to women with a psychotic spectrum disorder. Also, this would widen their social network and improve their self-esteem, which would enhance their quality of life too.

Also, the findings of this research highlight the urgent need to further explore the influence of sex on self-stigma and personal recovery. It has been evident that there are other sociocultural variables, such a migration status or the place of residence, that need to be studied to accomplish a more profound understanding of this dynamic, as they could be playing a role in it (22). If we could understand its underlying mechanisms, we could design more precise and individualized interventions for each person. This would represent an important improvement in numerous aspects of their lives.

Regarding the limitations of the present study, first, the sample size was conditioned by the availability of the different centers, so generalization of the results may be limited. In addition, we only explored the dynamic between self-stigma, personal recovery and sex, although it is clear that there are many factors at play. Another limitation would be that the data collection was made only by objective self-report scales and, even though this brings very valuable information about our study topic, we cannot completely comprehend it without delving deeper into the phenomenology.

For future research, we would recommend expanding the sample and exploring other factors, such as the cognitive and clinical insight, that could influence the process of self-stigma and personal recovery. Additionally, it would be important to complement the psychometric measures with ones that allow for a deeper dive into the perspective of the population, such as a focus group or interviews. Moreover, the collection of data in the post-treatment and the follow-up evaluations would allow us to see the evolution and changes of this interaction over time.

Given that gender roles and expectations can influence how individuals perceive themselves, relate to others, and seek or respond to treatment, future research would greatly benefit from adopting a gender-informed perspective. This approach could help deepen our understanding of how gender identity and internalized social norms intersect with self-stigma and personal recovery. Such a line of inquiry may yield valuable insights into the diverse experiences of individuals and contribute to the development of more personalized and effective mental health interventions.

In conclusion, the results of this study point out that sex has an important role in the development of the relationship between self-stigma and personal recovery. It is important to consider the self-stigma of a person with a psychotic spectrum disorder when trying to improve their personal recovery trajectory. Furthermore, it is imperative that we acknowledge the undeniable significance that sex has in this interplay and it is fundamental to incorporate it into research, policies and interventions.

## Data Availability

The raw data supporting the conclusions of this article will be made available by the authors, without undue reservation.

## ReCoMet Group

The ReCoMet Group is a multidisciplinary group of researchers who have actively collaborated in the development of this study (no ref. NCT06423651), all of them belonging to the different participating centers: Aguilera MI, Aldekoa A, Aznar A, Barajas A, Bassolas L, Birulés I, Blanco S, Casanovas C, Castellano X, Cazorla S, Cid J, Cobo J, Crespí C,  Cristeto S, Crosas JM, Díaz L, Frigola-Capell E, Garrido G, González A, González-Higueras F, Grossi A, Guillamet G, Jimeno T, Jordà A, Jurado L,  Korniyenko M, Layunta I, López-Botet M, López M, López T, Lozano L, Maestre A, Martínez I, Múñoz A, Muñoz N, Naives N, Navarro JB, Ochoa S, Palma-Sevillano C, Peniza M, Pérez R, Piña MD, Pijuan L,  Puidellívol N, Ramírez I, Rico J, Sastre-Buades A, Segarra A, Seseña LP, Sevilla-Llewellyn-Jones J, Schorr AL, Toraya AI, Torras R, Torres P, Traid V, Vallina O, Vázquez G, Verdaguer M,  Vidal C, Vidiella M, Zuzama N, Lamarca M. 
